# Policy analysis of the Iranian Health Transformation Plan in primary healthcare

**DOI:** 10.1186/s12913-019-4505-3

**Published:** 2019-09-18

**Authors:** Leila Doshmangir, Esmaeil Moshiri, Hakimeh Mostafavi, Minoo Alipouri Sakha, Abraham Assan

**Affiliations:** 1 Social Determinants of Health Research Center, Iranian Center of Excellence in Health Management, Health Management and Safety Promotion Research Institute, Tabriz University of Medical Sciences, Tabriz, Iran; 2 Department of Health Services Management, Tabriz Health Services Management Research Centre, School of Management and Medical Informatics, Tabriz University of Medical Sciences, Daneshgah St, Tabriz, 5165665811 Iran; 3 Social Determinants of Health Research Center, Semnan University of Medical Sciences, Semnan, Iran; 4 Health Economy, Standard and Health Technology Office, Shahid Beheshti University of Medical Sciences, Tehran, Iran; 5 Department of Global Health and Public Policy, School of Public Health, Tehran University of Medical Sciences, Tehran, Iran; 6 Global Policy and Advocacy Network (GLOOPLAN), Accra, Ghana; 7 Ghana College of Nurses and Midwives (GCNM), Accra, Ghana

**Keywords:** Primary health care, Policy analysis, Universal health coverage, Health reform, Health transformation plan, Iran

## Abstract

**Background:**

Health systems reform is inevitable due to the never-ending changing nature of societal health needs and policy dynamism. Today, the Health Transformation Plan (HTP) remains the major tool to facilitate the achievements of universal health coverage (UHC) in Iran. It was initially implemented in hospital-based setting and later expanded to primary health care (PHC). This study aimed to analyze the HTP at the PHC level in Iran.

**Methods:**

Qualitative data were collected through document analysis, round-table discussion, and semi-structured interviews with stakeholders at the micro, meso and macro levels of the health system. A tailored version of Walt & Gilson’s policy triangle model incorporating the stages heuristic model was used to guide data analysis.

**Results:**

The HTP emerged through a political process. Although the initiative aimed to facilitate the achievements of UHC by improving the entire health system of Iran, little attention was given to PHC especially during the first phases of policy development – a gap that occurred because politicians were in a great haste to fulfil a campaign promise.

**Conclusions:**

Health reforms targeting UHC and the health-related Sustainable Development Goals require the political will to improve PHC through engagements of all stakeholders of the health system, plus improved fiscal capacity of the country and financial commitments to implement evidence-informed initiatives.

**Electronic supplementary material:**

The online version of this article (10.1186/s12913-019-4505-3) contains supplementary material, which is available to authorized users.

## Background

A well-structured health system is important for sustainable health development [1]. Yet, no matter the robustness of a health system, reform is inevitable due to the need to respond to never-ending changing nature of societal health demands and policy dynamism [2, 3]. Despite its complex nature [4], countries worldwide constantly embark on health reforms to facilitate the achievement of universal health coverage (UHC) – by ensuring that all people (especially the vulnerable) can have access to the health services they need without risk of financial ruin or impoverishment [5, 6].

To attain UHC, health systems ought to be effective and efficient [7]. In Iran, the Ministry of Health and Medical Education (MoHME) is the largest governmental body. The institution is responsible for health policy formulation, resource mobilization, monitoring and evaluation, and regulating health service delivery throughout the health structure.

Main sources of revenue for the health system include: public (governmental) funds (main source), value-added tax (VAT), and direct out of pocket. The Social Security Organization (SSO) and Iran Health Insurance Organization (IHIO) are the main health purchasers in the country [8, 9]. Initially, IHIO was under the Ministry of Cooperative, Labour, and Social Welfare, until recently (in the year 2017) transferred to the MoHME. IHIO provides basic health insurance coverage for beneficiaries including government employees and their dependents, the self-employed and their dependents, rural residents, students and other professional associations [10]. The SSO is the largest single purchaser of healthcare, providing direct coverage to insured clients through 67 accredited hospitals and 270 clinics, across the country [8]. In addition, there are approximately 840 clinics, 670 hospitals and over 28,500 doctors and dentists that render services to clients, on a contract basis. PHC services are financed and provided by the government through a widespread PHC network [11].

The Iranian Primary Healthcare (PHC) system has undergone many policy reforms – efforts aimed to address major health system gaps, especially regarding access to health and distribution of health workforce [12]. Reforms include, the enactments of the National Health Service Corps Law (1964) [13], the expanded program on immunization and family health worker training program and the local midwifery training program in 1983 [14]. In 1985, the national health network [15] was also initiated to help increase coverage and access to health care and focusing on the prevention of communicable diseases [13]. Recent interventions include the establishment of the rural Family Physician (FP) program (2005), the pilot implementation of the rural insurance (Rural FP program) [16], and the urban FP program in 2010 which targeted selected cities with population of less than 50,000 [11]. Throughout these periods, emphasis was also placed on achieving quality health services and effective referral system. Major achievements (which have been attributed to a well-performing PHC system) [17], include the decline in population growth from about 3.9% during 1976–86 to 1.3% during the period of 2006–2011. Again, total fertility rate decreased from 7.0 children to below-replacement-level during the same period. Generally, life expectancy has also increased from 66 to 78 years during the period of 1990–2013 [18].

Following several policy debates and lessons from Turkey [19] and Thailand [20], the Government of Iran launched the “Health Transformation Plan” (HTP) in May 2014, to facilitate the attainments of UHC. Specific aims of the reform include: 1) increasing universal health insurance coverage 2) ensuring financial protection of patients 3) ensuring fair and equitable distribution of physicians and subspecialists throughout the country 4) improving hoteling and renovation in public sector 5) expanding outpatients services in the public health sector 6) promoting normal vaginal delivery (NVD) and preventing the increasing number of unnecessary caesarian sections 7) improving care and financial protection of patients with special needs and end stage diseases and 8) establishing air ambulance services [21]. The first phase of HTP focused on curative component of health, and targeted hospitals that were affiliated to the MoHME. The Plan was later extended to cover PHC services in November 2014 [22].

The PHC reform initiated include programs such as: the developments of appropriate structure of the health team, developing family practice, service delivery and PHC services in rural areas and cities with a population of under 20,000 and in suburban areas and cities with a population of about 20,000 to 50,000, integration of new services including smoking cessation, improving nutrition, preventing traffic accidents, promoting physical activity, preventing cancers, preventing cardiovascular disease, preventing diabetes, improving oral health and preventing mental illness and improving the health status of people with mental illnesses, establishing and strengthening intersectoral collaboration, and establishments and modification of FP and referral system. Other FP projects targeted the county and include review of training syllabus for health staff, developments of new academic disciplines and on the-job training, designing and implementing graduate courses including a Master of Family Medicine (an online modular course) and a family medicine specialty programme, an online electronic information system; a monitoring and evaluation system for services, and extensive assessment of clients’ satisfaction [23].

Overall, the implementation of HTP has contributed enormously in improving patients’ satisfaction, access to health care services, and has helped decrease out-of-pocket health expenditure [24]. Yet key challenges persist especially at the PHC level [22] – renewing the need for research on topics that can inform the health care reform debate especially at the local levels. This paper shed limelight on the recent health system reform in Iran, i.e., the HTP, and from the analysis described how the PHC was reorganized within the HTP. To achieve study objectives, we used the Walt & Gilson’s policy triangle [25] and the Stages Heuristic [26] model to guide analysis and interpretation of data.

We envision findings can help inform current and future policy action to accelerate progress of PHC system in Iran, and perhaps many other low and middle-income countries (LMICs) aiming to achieve UHC through the PHC approach.

## Methods

We employed the qualitative study design (using multiple methods) in exploring the phenomenon. Interviews and document analysis were the main sources of data – serving as “a confluence of evidence that breeds credibility” [27].

We conducted information-rich interviews with stakeholders – mainly people with improved knowledge of the health system evolution of Iran and the HTP. They include: representatives from universities, researchers, academics, and policy makers at the micro, meso and macro levels at the MoHME, health insurance organizations, Iran Medical Council, Parliament and Academy of Medical Sciences. A total of 23 participants were selected for the interview through purposive and snowball sampling techniques. The data obtained were again presented to a round-table discussion (constituting 12 senior policy makers), to enhance the validity and comprehensiveness of the study. A pre-tested interview guide was used. Questions covered issues pertaining to: policy content, context, stakeholders’ engagement, and policy making processes of the HTP at the PHC level. We also examined how the policy entered the policy agenda, its formulation, implementation, monitoring and evaluation. Face-to-face semi-structured interviews lasted at least 40 min, while the round-table discussion lasted approximately 2 h. In addition, we reviewed national documents (both printed, and electronic, i.e., data on national websites) (Additional file 1).

**Fig. 1:**
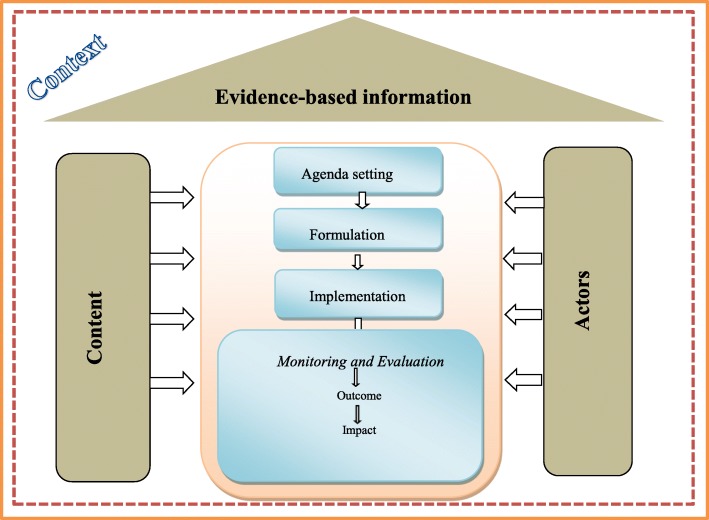
Conceptual interpretation of findings

All interviews and discussion were audio-recorded and transcribed verbatim. Coding was done using software MAXQDA 12. Codebooks were developed and themes that emerged from the data were repeatedly reviewed using research questions. Following the deductive framework approach [28], we used the policy stages model and policy triangle framework [22, 25] to understand how the policy emerged and unfolded at the PHC level – by highlighting how the policy content, context, and actors interacted during the policy processes, which include agenda setting, policy formation, implementation, and evaluation, to influence change (Fig. 1).

The policy triangle framework enabled us to explore different perspectives of the HTP, regarding how the content of policy, actors, context and processes interacted to shape the policy. Moreover, merging it with the stages heuristic model, we were able to explore the nonlinearity of practical decision-making processes including problem identification and agenda setting, policy formation, adoption, policy implementation, policy monitoring and evaluation. Besides, these two models have been widely used to analyze several health sector reforms in different countries [29–31], and remain applicable to the Iranian context, and useful in achieving study objective (Fig. 1).

## Results

Findings are categorized based on the stages of the policy process, and are explained below:

### Problem identification and agenda setting

Despite the numerous achievements of the PHC network programs, meaningful shortcomings existed. Most predominantly, there were conflicts of interest among stakeholders regarding values to guide the selection of potential public health issues to be considered in the policy agenda – i.e. the changes in diseases pattern and unmet health needs, the urgency to expand the service package of the PHC programs, and the inflexibility of existing initiatives to respond to the changing needs were all major problems that politically powerful groups were dissatisfied of, and demanded solutions to them.

“*… urbanization and the need to enhance community participation and inter-sectoral approach to addressing health needs were some of the pressure points for change – a change that could empower the public to take care of themselves against health problems, especially within the urban settings” (National health policy maker).*

There was the need for Iran to revamp its health system after the 8 year war with Iraq (1980–1988). However, governments were more focused on curative care while little attention was given to public health and preventive interventions. This was fascinated by the passing of laws to build hospitals across the country.

Following several reforms, Dr. Hassan Rouhani, the most recent President of Iran, placed health at the center of his campaign messages. Soon after taking office and in fulfillment of his campaign promise, the HTP was launched, and apparently became the most important social project of his administration. Areas of focus included: curatives care, public health and PHC, medical education and improving the medical pricing system.

“*The inadequate financial commitment to health by the previous governments has also contributed to the present challenges faced by the health system of Iran. In fact, the change in government and the political will of this government (the 11th Government of Iran) really triggered the reform*” *(*A senior national finance official*).*

Certain individuals, and institutional factors (e.g. social, religious, and economic factors) facilitated the problem entering the policy agenda. A major example is the statement released by the Supreme Leader of Iran, emphasizing the need for a comprehensive approach to health, and to be backed by laws. Again, existing national initiatives such as the FP and rural insurance program provided the needed foundation to improve the PHC system. The 4th and 5th five-year social, cultural and economic development plans also enhanced the implementation of FP, and provided the platform to further improve the referral systems. The implementation of targeted subsidies law (2013) which ensured the allocation of 10% of targeted subsidies to the health system also boosted resource allocation to the reform.

### Policy formulation

The policy formulation strategies aimed to achieve the highest level of precision. Experts with diverse background and experiences were recruited to design the reform. Evidence informed program (about 15 national programs and 10 projects) were discussed and approved following several meetings held by deputies of medical universities in the country. However, it was 4 months after the start of the HTP that priority was given to health promotion and preventive services for the underserved rural and border cities. Necessary steps were also taken to increase the number of health workforce and capacity building programs, continuation of existing pilot projects including the FP program in Fars (a province in the southwest Iran) and Mazandaran (a province in the north of Iran), and the nationwide expansion of FP and self-care programs.

*“This project was found in the 5*^*th*^ *Development Plan. Health indicators were presented to the Supreme Leader, and based on his recommendation a group was formed to ensure that the policy is enacted or brought into force by legislation” (*National senior policy maker*).*

### Policy implementation

Policy implementation at the PHC level was carried out in a stepwise approach by the Public Health Deputy of the MoHME. To minimize administrative and implementation flaws, policy makers capitalized on previous experiences and outcomes of the expansion of PHC to the cities. Yet, experts expressed their fears about the approach and the swift manner in which the policy was implemented. To them, several existing challenges including inadequate infrastructure, gaps in the referral system, and the lack of physician at health houses, ought to have been addressed first.

Moreover, ambiguities in several contracts between health care providers and their managements hindered the full realization and intended purpose of HTP. For example, regarding outsourcing and payments mechanisms, any delay in payments to the contracted companies often led to a delay in payments to service providers, thereby affecting the effectiveness and quality of services delivered. There were other legal objections to concession contracts which also hampered coherent implementation of the program in several cities.

*“The ideal way would have been to consult as many experts as possible, and to seek the opinions of the public about the various aspects of the HTP, followed by a pilot project, and then a scale-up if appropriate and based on the outcome of the pilot phase”. (*Health Researcher*).*

### Policy monitoring and evaluation

Monitoring and evaluation structures were designed to ensure effective and efficient implementation of HTP. For example, surveillance systems were revised to meet the objectives of the reform at the PHC level. Moreover, several universities and research centers were tasked to supervise the program and to prepare regular appraisal reports to track progress. Again, there were other ad hoc working groups within the Interior Ministry, Supreme Audit Court of Iran and the Parliament, to examine the policy content, its implementation and impact.

“*Public health committee review performance reports sent by subsidiaries during periodic meetings chaired by the Public Health Deputy of MoHME. Public officials and sometimes the minister of health do participate in the meetings to monitor the formulation and implementation of HTP at the PHC level*” (HTP Policy Document).

## Discussion

Governments of Iran have adopted several initiatives aimed at improving the health and wellbeing of the people, particularly, the poor and vulnerable. Recently, the HTP was initiated primarily to facilitate the attainments of UHC in Iran. Despite its numerous successes, delivering on this goal has been challenging given the substantial health system weaknesses, including gaps in providing PHC services to populations with limited access to health care. Although studies examining this phenomenon is not lacking, there has been much less attention given to how theories best informed analysis. To contribute to bridging such gap, we used the policy stages model [25] and policy triangle framework [22] to demonstrate how the reform emerged and unfolded in PHC.

While the use of stages heuristic framework yielded more variation and comprehension of public policy processes – i.e., covering different aspects of the complex and multi-dimensional components of agenda setting, formulation, implementation and evaluation, the policy stages model also enhanced our analyses of the contextual factors – including economic and political factors that influenced the policy and the process, and the objectives of the policy and the actors involved in decision-making.

Policymakers struggle to set priorities appropriately, particularly due to lack of evidence on which their decisions should be based. According to our study, the first stage of the policy process, i.e., where societal problems rise to the attention of decision makers (agenda-setting), problems such as unaccomplished healthcare needs of the population and limited availability and utilization of some public health services, which formed the basis for the policy reform, where not adequately prioritized. The agenda-setting phase is critical in policy development due to its subsequent effects on the policy process and its expected outcome [32, 33]. That is, the sustainability of any initiative largely depends on how well it is being guided by evidence [34, 35]. Commenting on such a challenge regarding the implementation of a voluntary health insurance in Lebanon, El-Jardali et al. [36] wrote that inability to provide adequate evidence for appropriate policy action may undermine its intended results.

Policy evolves over time and goes through iterations of stages as changes occur in the context of the policy issue. Contextual factors such as political and social issues can influence policy-making processes [37]. Based on our findings, the reform was fascinated by a strong political will, however, without thorough consultation – even with social institutions that were resisting change. Although achieving UHC necessitates strong political will [38] and the fiscal capabilities of a country, we anticipate that politicians were also in a haste to fulfil their campaign promise, without apprehending the consequences of their inability to thoroughly engage with stakeholders especially at the primary health care level. Moreover, it could be that, the four-year term of office is quite short, and politicians may want to focus on initiatives that would yield prompt results [39], including hospital-based reforms [40, 41], to the neglect of public health and PHC services – although delivery of services through the PHC approach could contribute to increase equity, efficiency and responsiveness [42, 43]. As argued broadly by Van De Bovenkamp et al. (2014), existing structure of policy actors (especially governments mechanisms) can affect healthcare [44]. Recognizing the role of stakeholders (including politically and economically influential groups and individuals) help facilitate progress in policy development and implementation [45, 46]. The unfinished agenda of the urban FP program after the change of the health minister in 2013, in Iran, is a typical example of how changes in leadership and key policy actors could change policy direction and its anticipated purposes [47].

Policies are products of a context within which they are developed – hence very useful to understand policy formation as a social and political process [48]. Our study revealed poor stakeholder engagement and public participation, focusing on PHC, during the implementation of HTP. Previous study by Moghadam (2012) has also shown ineffectiveness in stakeholders’ participation at the PHC level and its negative consequences on policy outcome [49]. Contrarily, a study conducted by Moshiri et al. (2016) in Iran disclosed poor policy outcome due to close relationship between top-level managers and frontline actors at the PHC level [12], despite the increasing evidence affirming the importance of community-based health workers (CHWs) (known as Behvarz in Iran) and the urban CHWs (Moraghebesalamat) in facilitativng the attainments of UHC through the PHC approach [50].

Although actors can have a negative impact on policy making processes, by either opposing an initiative or by acting to satisfy their personal interest [51], many health policy makers wrongly focus on the content of the reform, and overlook the role of actors in decision making – as argued by Walt and Gilson [52]. Achieving the aims of HTP at the PHC level demands robust FP program, with improved referral system. However, considering the complexities of the HTP within the PHC and its existing gaps, systematically evaluating the policy (across the different levels of the health system) could yield intended outcome. Specifically, consistent evaluation of PHC services using appropriate indicators can help meet the changing health needs and expectations due to changing demographics, disease burden, and issues of urbanization [53], undermining the development of high-quality and comprehensive PHC in Iran. Although, PHC has enormous potential to facilitate the attainment of UHC and a sustainable health system development [54–56], engaging independent organizations in result-based monitoring and evaluation system could yield improved outcome [57].

### Strengths and limitation of the study

The strengths of the study lie in its rigorous methodology.

- First, the conceptual framework used was able to accommodate wide range of perspectives, including social, political, and economic factors, for practical interpretation of policy development.
- We also enhanced the trustworthiness of the study through member checks, method and theory triangulation, and addressed reflexivity by involving researchers with different backgrounds.

Nonetheless, certain limitation exists – we are not certain of interviewees’ political, institutional and societal commitments to the initiative, which, we anticipate could influence the information they provided. In view of that, we recommend our findings to be interpreted with caution.

## Conclusions

Health systems reform is increasingly becoming relevant due to changing health needs and policy dynamism. The HTP remains the major instrument facilitating the achievements of UHC in Iran. Although the reform aimed to improve the entire health system of Iran, little attention has been given to the PHC – a gap fascinated by hasty policy implementation by politicians to fulfil a campaign promise.

Health reforms targeting UHC and the 2030 health-related Sustainable Development Agenda require the political will (including the financial commitments and improved fiscal capacity of the country) to implement evidence-informed initiatives targeting developments of PHC. Nevertheless, much can be realized when actors across all levels of the health system (especially at the primary and community levels) are thoroughly engaged throughout the policymaking processes.

## Additional file

Additional file 1: Interview guide. (DOCX 17 kb)

## Data Availability

The datasets used and/or analysed during the current study are available from the corresponding author on reasonable request.

## Abbreviations

FP: Family Physician
HTP: Health Transformation Plan
MoHME: Ministry of Health and Medical Education
PHC: Primary health care
UHC: Universal Health Coverage
WHO: World Health Organization
