# Supplementary material for: Policy analysis of the Iranian Health Transformation Plan in primary healthcare
Source: BMC Health Serv Res. 2019 Sep 18;19:670. doi: 10.1186/s12913-019-4505-3 (PMC6751681; doi:10.1186/s12913-019-4505-3)
Supplement: Supplementary file 1 — Interview guide. (DOCX 17 kb) [file 12913_2019_4505_MOESM1_ESM.docx]

**Additional File 1: Interview guide**

Date and venue

Name and position of interviewee

1. Please, can you share with us your general opinion about the Health Transformation Plan in Iran?
2. What were the major reasons behind the formulation and implementation of this reform at the PHC level?
3. How did the reform in PHC enter into the policy agenda, and by whom (individuals, groups or organizations)?
4. In your opinion, how well did the reform cover issues pertaining to the PHC of the country?
5. How was the reform in PHC formulated and implemented? Were all stakeholders in support of the reform in this level? Please explain in detail how well the initiative has been accepted by the actors involved.
6. Is there any financing model to ensure the sustainability of the reform at this level?
7. To what extent do you think the reform was clear in content, context, processes of implementation and monitoring, and the expected results and outcomes/impacts)? Kindly explain while you place emphasis on the PHC.
8. To what extent do you think initiatives at this level were guided by evidence?
9. Can you please share with us (with evidence) the overall impacts (positive and negative) of the reform at the PHC level and community?
10. So far, what are the feedbacks you have received from policy-makers or stakeholders prior to the implementation of the reform? Please discuss. How did you deal with those feedbacks?
11. How well has the reform been successful in attaining its intended outcome at the PHC level? Kindly explain with evidence.
12. Please, can you share with us the strengths and challenges faced? When given the opportunity the make amendments, which aspects of the reform would you change or improve? Can you explain in detail the reasons for your decision?
13. Last but not least, is there any information relevant to the scope of our topic which we have not discussed? Please feel free to share.

Many thanks for your invaluable contribution.
